# Bedside calculation of mechanical power during volume- and pressure-controlled mechanical ventilation

**DOI:** 10.1186/s13054-020-03116-w

**Published:** 2020-07-11

**Authors:** Davide Chiumello, Miriam Gotti, Mariateresa Guanziroli, Paolo Formenti, Michele Umbrello, Iacopo Pasticci, Giovanni Mistraletti, Mattia Busana

**Affiliations:** 1 SC Anestesia e Rianimazione, Ospedale San Paolo – Polo Universitario, ASST Santi Paolo e Carlo, Via Di Rudinì, 8, 20142 Milan, Italy; 2 Dipartimento di Scienze della Salute, Università degli Studi di Milano, Milan, Italy; 3 Centro Ricerca Coordinata di Insufficienza Respiratoria, Milan, Italy; 4 Dipartimento di Fisiopatologia Medica Chirurgica e dei Trapianti, Università degli Studi di Milano, Milan, Italy; 5 Department of Anesthesiology, Emergency and Intensive Care Medicine, University of Göttingen, Göttingen, Germany

**Keywords:** Mechanical power, Mechanical ventilation, Ventilator-induced lung injury, Acute respiratory distress syndrome, Lung protective ventilation, Acute respiratory failure, Driving pressure, Lung protection, Respiratory failure, Respiratory rate

## Abstract

**Background:**

Mechanical power (MP) is the energy delivered to the respiratory system over time during mechanical ventilation. Our aim was to compare the currently available methods to calculate MP during volume- and pressure-controlled ventilation, comparing different equations with the geometric reference method, to understand whether the easier to use surrogate formulas were suitable for the everyday clinical practice. This would warrant a more widespread use of mechanical power to promote lung protection.

**Methods:**

Forty respiratory failure patients, sedated and paralyzed for clinical reasons, were ventilated in volume-controlled ventilation, at two inspiratory flows (30 and 60 L/min), and pressure-controlled ventilation with a similar tidal volume. Mechanical power was computed both with the geometric method, as the area between the inspiratory limb of the airway pressure and the volume, and with two algebraic methods, a comprehensive and a surrogate formula.

**Results:**

The bias between the MP computed by the geometric method and by the comprehensive algebraic method during volume-controlled ventilation was respectively 0.053 (0.77, − 0.81) J/min and − 0.4 (0.70, − 1.50) J/min at low and high flows (*r*^2^ = 0.96 and 0.97, *p* < 0.01). The MP measured and computed by the two methods were highly correlated (*r*^2^ = 0.95 and 0.94, *p* < 0.01) with a bias of − 0.0074 (0.91, − 0.93) and − 1.0 (0.45, − 2.52) J/min at high-low flows. During pressure-controlled ventilation, the bias between the MP measured and the one calculated with the comprehensive and simplified methods was correlated (*r*^2^ = 0.81, 0.94, *p* < 0.01) with mean differences of − 0.001 (2.05, − 2.05) and − 0.81 (2.11, − 0.48) J/min.

**Conclusions:**

Both for volume-controlled and pressure-controlled ventilation, the surrogate formulas approximate the reference method well enough to warrant their use in the everyday clinical practice. Given that these formulas require nothing more than the variables already displayed by the intensive care ventilator, a more widespread use of mechanical power should be encouraged to promote lung protection against ventilator-induced lung injury.

## Introduction

According to a recent international observational study, acute respiratory distress syndrome (ARDS) is reported in up to 10% of total intensive care admissions and 20% of all patients who require mechanical ventilation [1]. Mechanical ventilation remains a cornerstone of ARDS management [2, 3] as it improves the severe hypoxemia and reduces the work of breathing. However, the mechanical forces (pressure, volume, and flow) generated by the interactions between the ventilator and the respiratory system can further damage the lung, a process known as ventilator-induced lung injury (VILI) [4]. To limit VILI, several recommendations based primarily on the tidal volume [5], driving pressure [6], and positive end-expiratory pressure (PEEP) have been put forward [7]. More recently, the inspiratory flow [8] and respiratory rate [9] have also been recognized as possible factors promoting VILI.

To combine all these elements in a single physical variable, Gattinoni et al. proposed the concept of mechanical power (MP) to estimate the contribution of the various ventilator-related causes of lung injury [10]. MP is the energy delivered to the respiratory system over time, which is the product of the absolute proximal airway pressure and related changes in volume and respiratory rate [10]. Animal data showed that increasing MP was associated with an increase in lung edema and lung damage [11–13]. Furthermore, two studies in patients with and without ARDS found that MP computed during the first days of mechanical ventilation was independently associated with mortality, which rose significantly above a certain level of MP [14, 15]. Nevertheless, although MP can be used at the bedside, it is rather cumbersome to be calculated, a factor that heavily hinders its use in clinical practice [8, 16, 17].

Two methods can be employed to calculate MP, a direct measurement, as the dynamic inspiratory area of the airway pressure and volume curve during the respiratory cycle (i.e., the geometric method), or using equations. One of the first equations proposed to compute the MP requires the knowledge of several elements such as the tidal volume, respiratory rate, elastance, resistance, and inspiratory time [10], variables for which an inspiratory hold is necessary in order to adequately distinguish between the resistive and the elastic components of airway pressure. In order to facilitate the calculation of MP, the original equation was proposed in a simplified form requiring only the measurement of airway plateau pressure, PEEP, and tidal volume. With the same aim, Giosa et al. [18] described an even simpler equation which did not require an inspiratory hold, trading a small degree of accuracy to allow the continuous display by the ventilator with no action from the clinician. However, both these equations can be used only during volume-controlled ventilation with constant inspiratory flow in sedated patients with no spontaneous respiratory drive. During pressure-controlled ventilation, in which the airway pressure is held constant, and the flow is decelerated during the mechanical breath, two alternative equations have been suggested [19, 20].

The aim of this study was to compare the geometric method to measure MP, considered the reference standard, with the algebraic formulas, both for volume and pressure-controlled ventilation in a group of sedated and paralyzed patients, to understand whether the easier to use surrogate formulas were suitable for the everyday clinical practice. This would warrant a more widespread use of mechanical power to promote lung protection.

## Materials and methods

### Study population

Patients admitted to the intensive care unit from January to June 2018 after elective surgery (excluding thoracic and cranial surgery) or with medical disease were considered eligible. The study was approved by the institutional review board of our hospital (Comitato Etico Interaziendale Milano Area A 1, n. 628), and informed consent was obtained according to Italian regulations. Exclusion criteria were hemodynamic instability (systolic arterial pressure < 100 mmHg) and COPD (Gold stages 3 and 4).

### Study design

Critically ill patients with acute respiratory failure, kept in supine position and sedated and paralyzed for clinical reasons, were ventilated in volume-controlled mode with a tidal volume of 6–8 mL/kg of predicted body weight with two inspiratory flows (high, 60 L/min, and low, 30 L/min), with a square waveform. Subsequently, pressure-controlled ventilation was applied to reach the same tidal volume as volume-controlled ventilation. PEEP and respiratory rates were not changed throughout the study.

### Measurements

The inspiratory flow rate was measured with a heated pneumotachograph (Fleisch no. 2, Fleisch, Lausanne, Switzerland). Airway pressure was measured proximally to the endotracheal tube with a dedicated pressure transducer (MPX 2010 DP, Motorola, Solna, Sweden). Flow and airway pressure were collected at a sampling rate of 100 Hz and stored for analysis (Colligo, Elekton, Milan, Italy).

The following variables were measured in each condition: tidal volume, inspiratory time, peak airway pressure, plateau airway pressure, and PEEP during an end-inspiratory and expiratory pause. Means were computed over five consecutive breaths.

### Computation of mechanical power

#### Geometric method

During volume- and pressure-controlled ventilation, the MP was *measured* as the energy per breath (the area between the inspiratory limb of the airway pressure curve and the volume (*y*-axis) (Figure S1), expressed in Joules and multiplied by the respiratory rate (J/min) [10, 21].

### Calculation of MP during volume-controlled ventilation

MP can be *calculated* with two algebraic formulas: a comprehensive and a surrogate. Overall, the equations described below have the aim to approximate the geometric area of the dynamic pressure-volume curve starting from the variables usually measured at the bedside. One general assumption that needs to be considered is that, in all these equations, the respiratory system is considered as one single compartment (i.e., with only one time constant, the product of resistances and compliance). In most of the cases, this seems a reasonable approximation of the reality.

#### Comprehensive formula

By “comprehensive,” we mean an equation that takes into account both the resistive and elastic components of the mechanical breath, giving a more accurate measurement of MP. It requires an inspiratory hold. This equation derives from a mathematical simplification of the original mechanical power formula [10], with the advantage that the calculation starts from the measured peak, plateau, and end-expiratory pressure, allowing a much faster bedside computation.

$$ \mathrm{MP}=0.098\bullet \mathrm{RR}\bullet {V}_t\bullet \left[\mathrm{Peak}\ \mathrm{inspiratory}\ \mathrm{pressure}-\frac{1}{2}\left(\mathrm{Plateau}\ \mathrm{pressure}-\mathrm{PEEP}\right)\right] $$

where 0.098 is a conversion factor from cmH2O l min^−1^in J/min, RR is the respiratory rate, and *V*_t_ is the tidal volume in liters. The term (Plateau pressure − PEEP) is the driving pressure. As stated in the original paper describing the formula [10], being mathematically identical to the original equation, it suffers from the same limitations: the compliance of the respiratory system is considered linear over the range of pressure and volumes considered.

#### Surrogate formula

We use the term “surrogate” to mean an equation that is easier to calculate than the comprehensive one, as no inspiratory hold is required. The rationale behind this simplified equation is to trade a certain degree of accuracy for ease of calculation at the bedside. The equation, recently proposed by Giosa et al. [18], aims to simplify the calculations offering an easy to remember equation mainly dealing with the problem of the airway resistances. In the original paper, a value of 10 cmH_2_O s l^−1^ has been considered sufficiently accurate to represent a general population of intubated and mechanically ventilated patients. Expressing that value in cmH_2_O min l^−1^ allows to lump together the term resistances (cmH_2_O min l^−1^) × inspiratory flow (l/min).

$$ \mathrm{MP}=\frac{\mathrm{VE}\bullet \left(\mathrm{Peak}\ \mathrm{pressure}+\mathrm{PEEP}+\frac{\mathrm{Inspiratory}\ \mathrm{flow}}{6}\right)}{20} $$

where VE is the minute ventilation expressed in l/min. Peak pressure and PEEP are expressed in cmH_2_O. Inspiratory flow/6 turns out to be also a pressure term, following the explanation above. Clearly, the more the true airway resistances of the patient are far from the hypothetical value of 10 cmH_2_O s l^−1^, the greater the error made. Nevertheless, in the original paper, even for values greater than 15 cmH_2_O s l^−1^, the bias remained reasonably low.

### Calculation of MP during pressure-controlled ventilation

The MP was calculated with a comprehensive and a surrogate formula.

### Comprehensive formula

$$ \mathrm{MP}=0.098\bullet \mathrm{RR}\bullet {V}_t\bullet \left[\mathrm{PEEP}+{\Delta  P}_{\mathrm{insp}}\bullet \left(1-{e}^{\frac{-{T}_{\mathrm{insp}}}{RC}}\right)\right] $$

where Δ*P*_insp_ is the pressure (cmH_2_O) above PEEP during pressure-controlled ventilation, *T*_insp_ is the inspiratory time (s), and *C* and *R* are the respiratory system compliance (ml/cmH_2_O) and resistances (cmH_2_O s l^−1^), respectively. Compliance and respiratory system resistance (airways and tissue resistances) were calculated during end-expiratory and end-inspiratory pauses from the data when the patient was ventilated in volume-controlled mode with an inspiratory flow of 30 L/min. The main assumption in this equation is that airway resistances are considered constant throughout the breath, which is not necessarily true, as resistances are also a function of flow, which varies during pressure-controlled ventilation.

#### Surrogate formula

$$ \mathrm{MP}=0.098\bullet \mathrm{RR}\bullet {V}_t\bullet \left[\mathrm{PEEP}+{\Delta  P}_{\mathrm{insp}}\right] $$

where 0.098 is a conversion factor from cmH_2_O l min^−1^ in J/min, RR is the respiratory rate, and *V*_t_ is the tidal volume in liters. PEEP and Δ*P*_insp_ is the pressure (cmH_2_O) above PEEP during pressure-controlled ventilation. This extremely simplified equation works under the assumption that the delivered pressure wave is perfectly squared. In the original paper, this assumption proved to hold reasonably well [19].

#### Statistical analysis

The MP measured by the geometric method and calculated with both the comprehensive and surrogate algebraic formulas were compared with the Bland-Altman technique and linear regression. The absolute error was calculated as (1.96*SD)/mean of the reference. A repeated measures ANOVA was used to establish the difference between the MP delivered in the volume- and pressure-controlled ventilation modes. *p* values at the post hoc analysis were adjusted with the Bonferroni correction, and two-tailed *p* values < 0.005 were considered statistically significant. Statistical analysis was done with Jupyter (Python 3.7) and the Pandas, Numpy, Statsmodels, and Pingouin packages.

## Results

Forty patients were enrolled. Table 1 reports their main clinical characteristics. The mean applied tidal volumes during volume-controlled ventilation at 30 and 60 L/min flow and pressure-controlled ventilation were 419 ± 68 mL (flow 30 L/min), 398 ± 73 mL (flow 60 L/min), and 422 ± 66 mL. Minute ventilation was 6.0 ± 1.3 L/min (flow 30 L/min), 5.7 ± 1.3 L/min (flow 60 L/min), and 6.0 ± 1.4 L/min. The differences in these measurements were not significant (*p* = 0.12 for tidal volume and 0.28 for minute ventilation). As expected, during volume-controlled ventilation, the calculated airway and tissue resistances were higher at 60 L/min (*p* < 0.001) as shown in Figure S2.

**Table 1:** Clinical characteristics of the study population

Variables	Population (*n* = 40)
Age (year)	71 ± 12
Height (cm)	167 ± 7
BMI (kg/m^2^)	25 ± 4.3
Intensive care unit stay (days)	3.8 ± 5.9
Medical/elective surgery (*n*)	11–29
PaO_2_/FiO_2_ (mmHg)	244 ± 135
PaCO_2_ (mmHg)	42.5 ± 4.8
Respiratory rate (bpm)	14.3 ± 1.8
Tidal volume (mL)	413 ± 70
Tidal volume/kg PBW (mL/kg)	7.6 ± 2.3
Minute ventilation (L/min)	5.9 ± 1.3
Clinical PEEP (cmH_2_O)	5.2 ± 0.9
Driving pressure (cmH_2_O)	7.1 ± 1.6
Respiratory system elastance (cmH_2_O/mL)	17.7 ± 4.3

### Volume-controlled mechanical ventilation

**Fig. 1:**
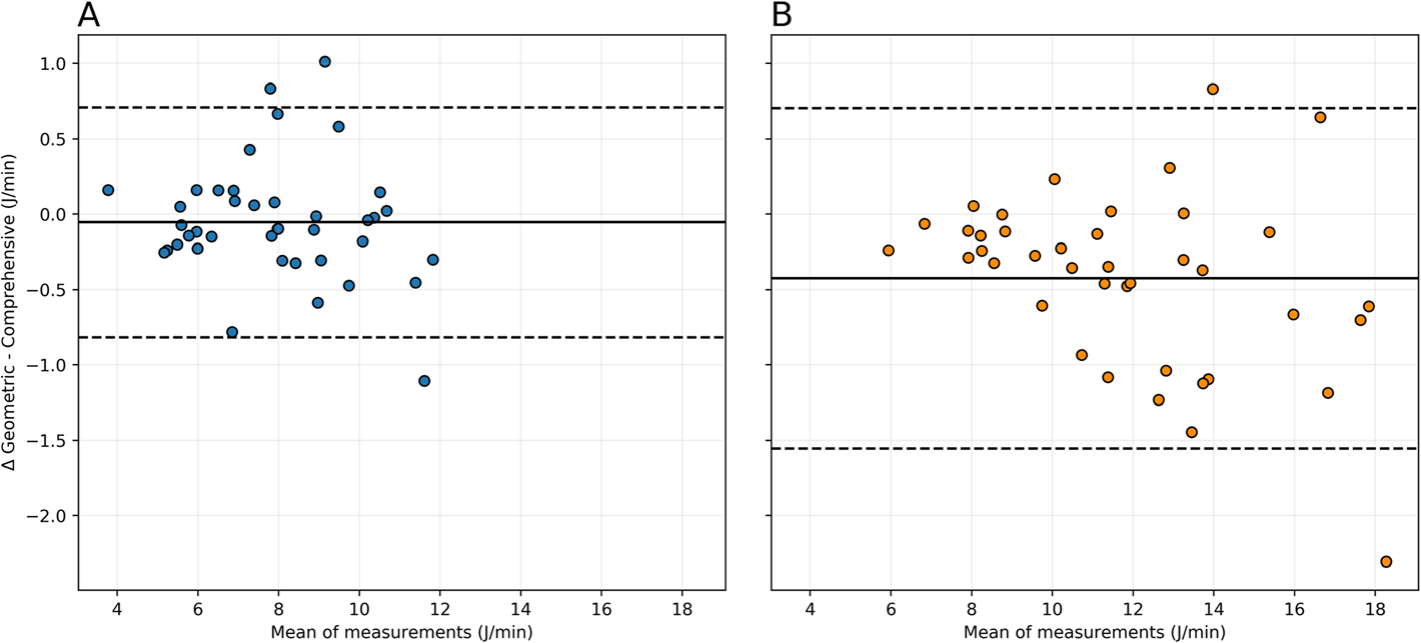
Bland-Altman analysis of the comprehensive equation for volume-controlled ventilation at 30 L/min (**a**) and 60 L/min (**b**) of inspiratory flow against the reference geometrical method

**Fig. 2:**
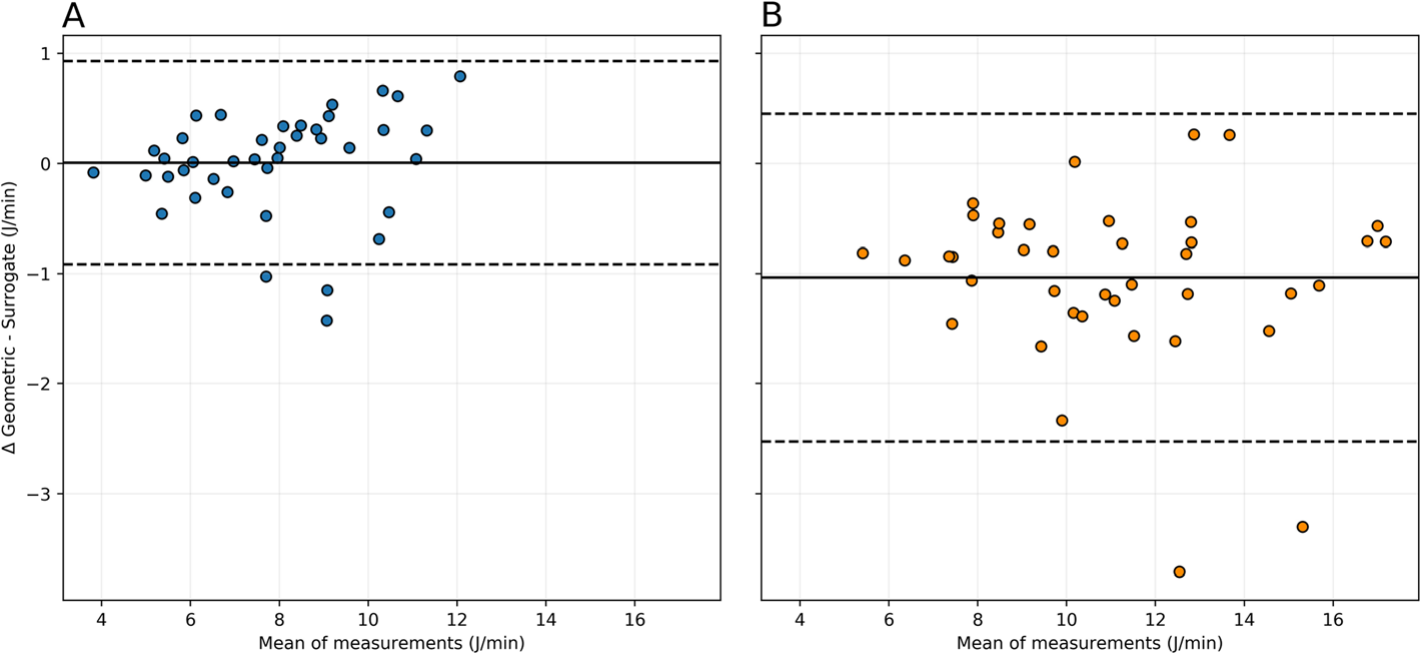
Bland-Altman analysis of the surrogate equation for volume-controlled ventilation at 30 L/min (**a**) and 60 L/min (**b**) of inspiratory flow against the reference geometrical method

The regressions between the MP assessed by the geometric method and the comprehensive algebraic formula for 30 and 60 L/min of inspiratory flow are shown in Figure S3. The two methods were closely correlated (*r*^2^ = 0.96, *p* < 0.001, and *r*^2^ = 0.97, *p* < 0.001). The Bland-Altman analyses are reported in Fig. 1. For an inspiratory flow of 30 L/min, the mean difference was − 0.053 J/min with upper and lower limits of agreement (± 1.96 SD) of 0.77 and − 0.81 J/min; for an inspiratory flow of 60 L/min, the mean difference was − 0.4 J/min with upper and lower limits of agreement of 0.7 and − 1.5 J/min. The MP calculated with the surrogate equation was also closely correlated (*r*^2^ = 0.95 and 0.94) with bias of − 0.0074 (0.91, − 0.93) and − 1.0 (0.45, − 2.52) J/min at low and high inspiratory flows (Fig. 2 and S4).

### Pressure-controlled mechanical ventilation

**Fig. 3:**
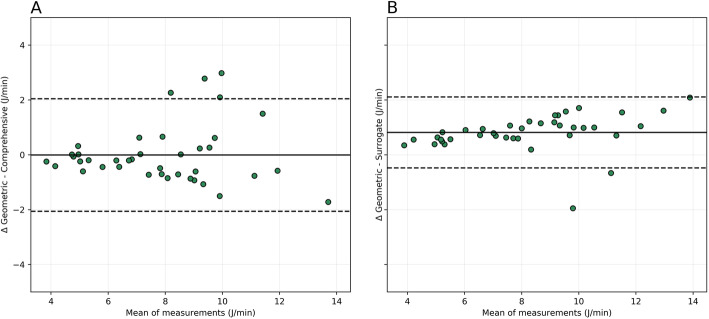
Bland-Altman analysis of the comprehensive (**a**) and the surrogate equations (**b**) for pressure-controlled ventilation against the reference geometrical method

The regression between the MP computed by the geometric method and comprehensive algebraic formula is shown in Figure S5, left panel. The two methods were closely correlated (*r*^2^ = 0.81, *p* < 0.001). The Bland-Altman analyses are reported in Fig. 3a. The mean difference was − 0.001 J/min, with upper and lower limits of agreement 2.05 and − 2.05 J/min.

The MP with the surrogate equation were closely correlated (*r*^2^ = 0.94, *p* < 0.001) with a bias of − 0.81 J/min (2.11, − 0.48 upper and lower limits of agreement) (Fig. 3b and S5, Panel B).

The mean values in each experimental set-up are summarized in Table S1.

## Discussion

The MP can be computed, as a gold standard, by analysis of the dynamic inspiratory airway pressure-volume curve, which measures the physical phenomenon (i.e., the geometric method). Alternatively, equations can be applied which assume that the respiratory system elastance and resistance are constant during inspiration [10]. Unfortunately, although the intensive care ventilator could in fact easily compute or measure the MP directly, this measurement is not automatically available, obliging physicians to calculate it manually. Normally, in the course of ARDS, mechanical ventilation is applied either in volume-controlled or in pressure-controlled mode [22]. The different characteristics of the flow curve in the two modes imply that it is not possible to apply the same equations for these two ventilatory setups.

### Volume-controlled ventilation

For volume-controlled ventilation, Gattinoni et al. originally proposed an equation that was complicated to compute at the bedside because it included all the respiratory physiological variables [10]. The same authors proposed also a simpler equation that, starting from the measured values of peak, plateau and end-expiratory pressure was mathematically identical to the original one, allowing to calculate MP with the data displayed by the ventilator, provided that an end-inspiratory hold was performed. We called this “comprehensive formula.” Recently, Giosa and colleagues [18] proposed a surrogate formula that allowed to calculate MP without the inspiratory hold. In their original paper, however, the equation was not tested against the reference geometrical method. The present study indicates that for volume-controlled ventilation, the MP measured by the comprehensive and the surrogate equations were highly correlated with the reference-standard geometric method, with limits of agreement mostly within 2 J/min. This advocates that the simpler surrogate formula is of high clinical utility, allowing to quickly calculate MP even in settings where performing an inspiratory hold is either not possible (operating theater, patient transport) or not feasible (elevated number of patients and/or staff shortage). Of note, the fixed value for resistances assumed by the surrogate equation quite likely underestimates the “real” MP at high inspiratory flow rates, as the calculated resistances tend to increase because of the turbulent motion component. However, Giosa et al. found that even doubling the resistance up to 20 cmH_2_O s/L, the underestimation bias remained quite low—in the order of 1.3 J/min. In the current study, to assess this surrogate method at two different resistances (high and low) directly against the geometric method, we applied high and low inspiratory flows (30 and 60 L/min). At both levels, the error between the geometric and the surrogate methods remained clinically negligible.

### Mechanical power at two levels of inspiratory flow

As shown in Table S1, both the measured (geometrical method) and the calculated (comprehensive and surrogate formulas) MP at 60 L/min was higher than at 30 L/min. This is not unexpected: as already stated by Gattinoni et al. in the original paper [10], MP increases with increasing airway resistances and flow. Indeed, at higher flows, not only the airway resistances, but also the tissue resistances increase, as the higher rate of tidal strain requires more energy. While it can be reasonably assumed that the energy dissipated in the airways and the endotracheal tube does not cause VILI, the one dissipated to perform the tissue strain has been already shown to cause damage and should not be ignored [23].

### Pressure-controlled ventilation

Until Becher et al. [19] and Van der Meijden et al. [20] reported two equations to measure the MP in pressure-controlled ventilation, it had been erroneously assessed with equations developed and validated only for volume-controlled ventilation. For example, Serpa Neto et al. [15] computed the MP in a big population of mechanically ventilated patients using a single formula independently of the actual type of ventilation. Van der Meijden et al. finally suggested a comprehensive formula that considers all the variables. The main drawback of this approach is the flow-dependency of the resistances: as flow is not constant in pressure-controlled ventilation, whatever value used is intrinsically approximated. Nevertheless, the need of an inspiratory hold, calculation of the resistances, and the presence of an exponential make this equation usable only if directly implemented in the ventilator. Becher et al. besides their original equation proposed an extremely easy to use surrogate formula for the same purpose. Interestingly, in our population, the surrogate performed equally if not better than the comprehensive formula from Van der Meijden, having a higher *R*^2^ and, despite a trivially larger bias, a smaller confidence interval. This discrepancy between the comprehensive and the surrogate formulas is probably due to the assumptions that need to be made to calculate the resistances during an inflation with decreasing flow. The clinical consequence is that the surrogate formula, despite its simplicity, has a high degree of accuracy and can be safely used at the bedside without the need of complex calculations.

### Clinical implications

Nowadays, plateau pressure and driving pressure are the de facto clinically used variables to monitor whether a certain mechanical ventilation setup is protective or not [6]. The main benefit of driving pressure is the extreme ease of the calculation that, together with its association with mortality, allowed a rapid adoption around the world. Nevertheless, we believe that mechanical power, also including the effects of respiratory rate, flow, and PEEP, gives a much more comprehensive view on the ventilator-related causes of VILI [24] but its widespread application has been hindered by the complexity of its calculation. We believe that our data do support the use of the surrogate formulas at the bedside to add a tile in the puzzle of lung protection. Clinicians are used to use low tidal volumes (6 mL/kg, driving pressure < 14 cmH_2_O), and to maintain a viable CO_2_ clearance, they ramp up the respiratory frequency. It is therefore of paramount importance to sensitize the intensivists to consider that high respiratory rate does injure the lung as it is part of the whole energy package delivered by the ventilator [11, 25].

### Limitations

This study has several limitations. First, the comprehensive and surrogate equations can be currently applied only in sedated and paralyzed patients with no active breathing. Second, it is reasonable to think that the *biological* effects of MP will be clearer once: (1) MP is partitioned to the lung only by using the transpulmonary pressure [26] instead of the total airway pressure and (2) it is normalized to the lung volume or to the amount of well-aerated lung tissue, as MP is an extensive property and might have different effects depending on the amount of lung mass bearing a given energy load [14].

## Conclusion

Both for volume-controlled and pressure-controlled ventilation, the surrogate formulas show enough accuracy to warrant their use in clinical practice. The use of the comprehensive formulas is encouraged, especially when time and staff availability allow to allocate more time to the management of patients. The geometrical method, considered the gold standard, should be reserved to a research and academic environment, unless directly integrated in the next-generation ventilator software.

## Supplementary information

**Additional file 1.**

## Data Availability

The dataset used is available upon a justified request.
