# Supplementary material for: Bedside calculation of mechanical power during volume- and pressure-controlled mechanical ventilation
Source: Crit Care. 2020 Jul 11;24:417. doi: 10.1186/s13054-020-03116-w (PMC7351639; doi:10.1186/s13054-020-03116-w)
Supplement: Supplementary file 1 — Additional file 1. [file 13054_2020_3116_MOESM1_ESM.docx]

**MECHANICAL POWER: BEDSIDE CALCULATION DURING VOLUME AND PRESSURE CONTROLLED VENTILATION**

**SUPPLEMENTAL MATERIAL**

## Supplemental methods


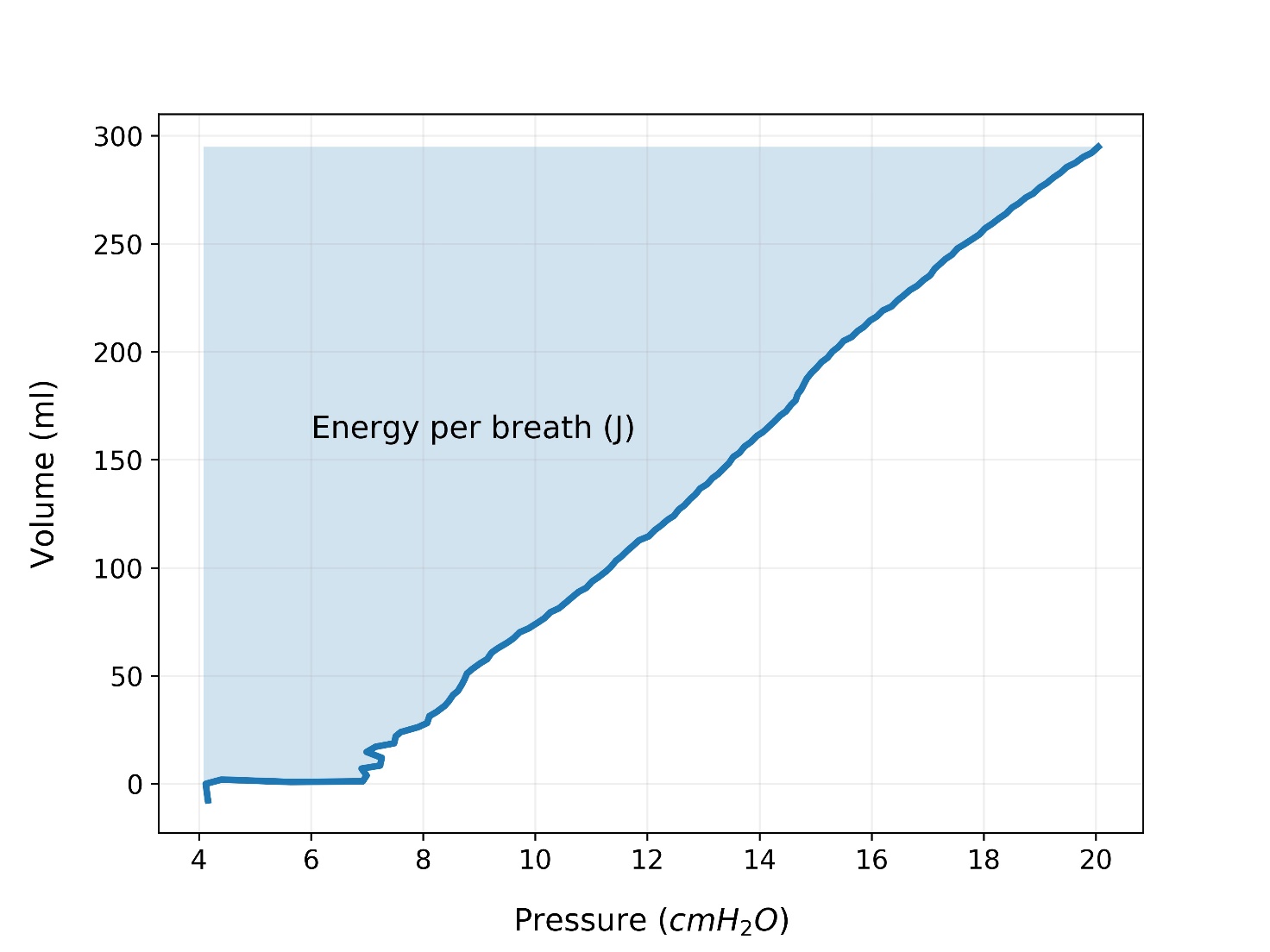


**Figure S1**: Geometrical method to calculate mechanical power. The value obtained as the area of the pressure-volume loop needs to be multiplied by the respiratory rate.

## Supplemental results

**
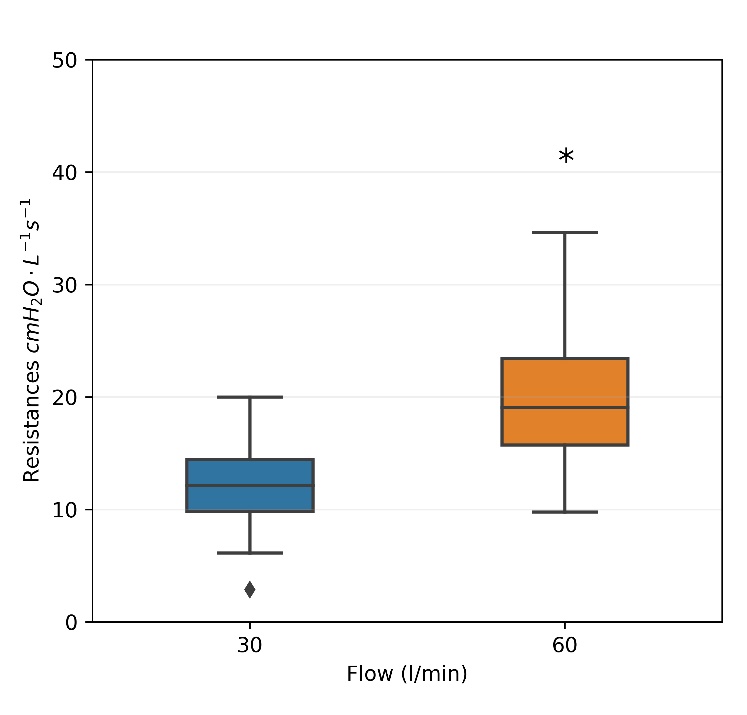
**

**Figure S2**: Airway resistances measured during volume-controlled ventilation at 30 and 60 l/min.

*) p < 0.05 compared to 30 l/min.

**
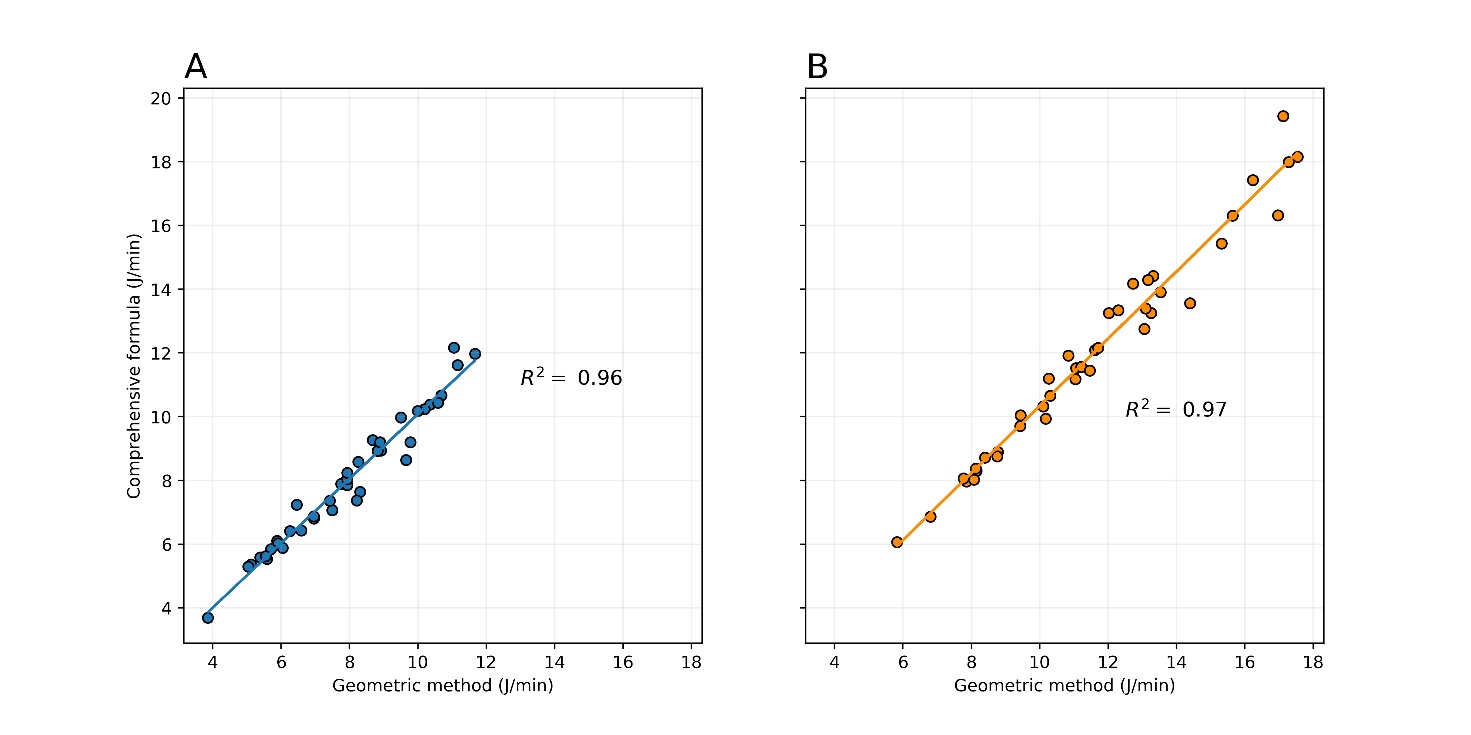
**

**Figure S3**: Linear regressions between the geometrical method and the comprehensive algebraic formula, for volume-controlled ventilation, at 30 L/min of inspiratory flow (Panel A) and 60 L/min of inspiratory flow (Panel B)

**
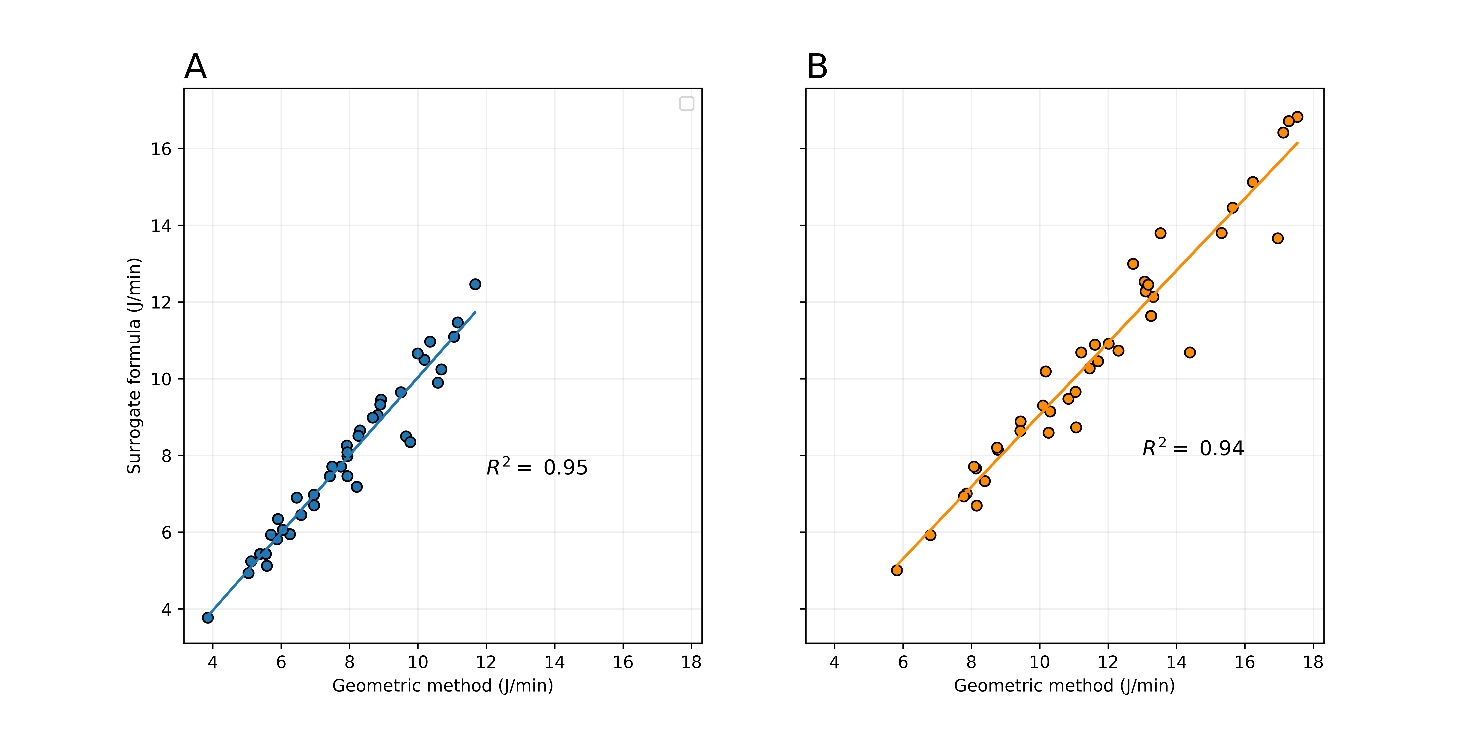
**

**Figure S4:** Linear regressions between the geometrical method and the simplified surrogate formula, for volume-controlled ventilation, at 30 L/min of inspiratory flow (Panel A) and 60 L/min of inspiratory flow (Panel B)

**
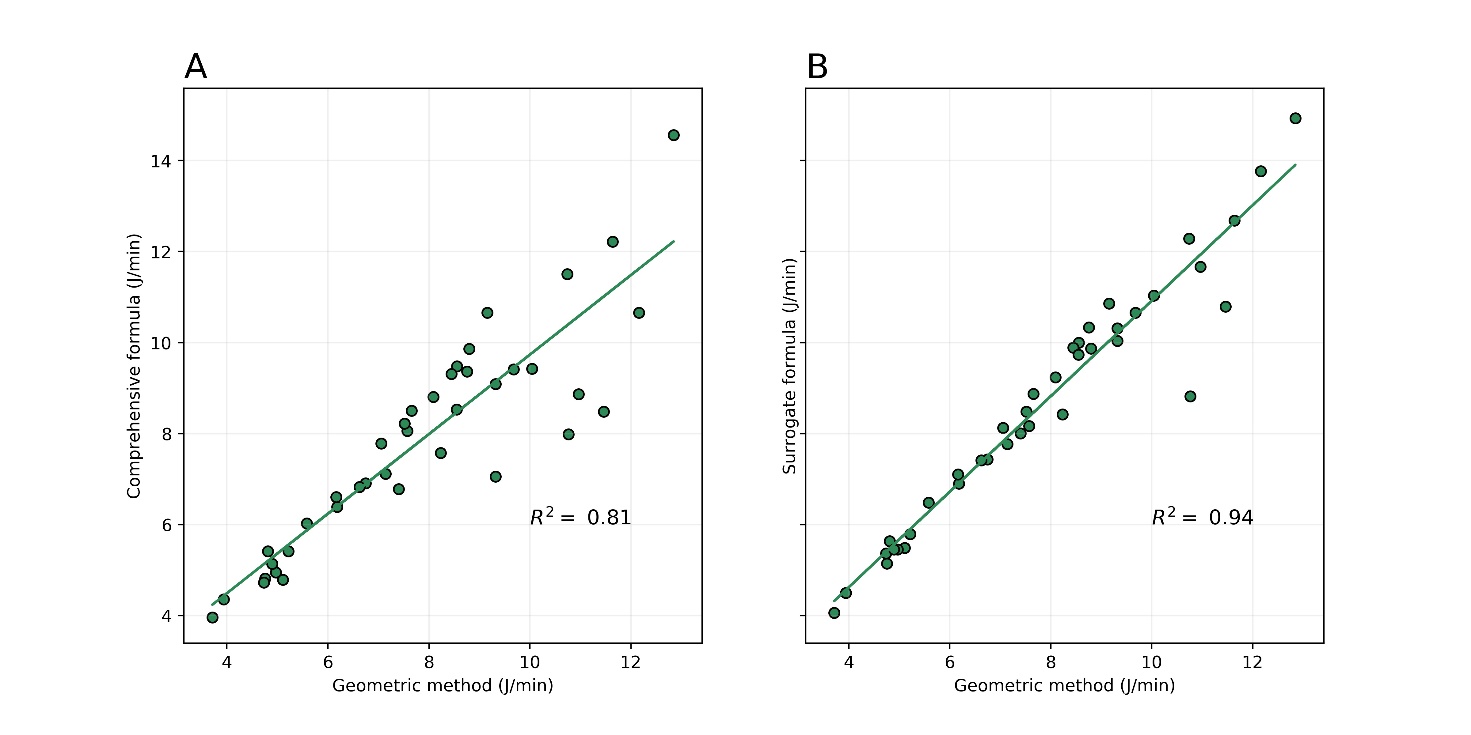
**

**Figure S5**: (Panel A): Linear regression between the geometrical method and the comprehensive formula proposed by Van der Meijden for pressure-controlled ventilation.

(Panel B): Linear regression between the geometrical method and the surrogate equation proposed by Becher for pressure-controlled ventilation.

|  | **VCV 30 L/min** | | | | **VCV 60 l/min** | | | | **PCV** | | | | |
| --- | --- | --- | --- | --- | --- | --- | --- | --- | --- | --- | --- | --- | --- |
|  | Geometric loop | Algebric | Surrogate | p value | Geometric loop | Algebric | Surrogate | p value | Geometric loop | Algebric | Surrogate | p value |  |
| **Mechanical power** | 7.91 ± 1.98 | 7.96 ± 2.04 | 7.91 ± 2.06 | 0.695 | 11.60 ± 3.08 **$£** | 12.02 ± 3.29 ***£** | 10.56 ± 2.99 ***$** | 0.01 | 7.84 ± 2.39 | 7.84 ± 2.35 | 8.64 ± 2.62 ***$** | 0.01 |  |

**Table S1**: Summary table of the mean ± standard deviation of the mechanical power values for each experimental setup.

* p < 0.05, different from Geometric loop

$ p < 0.05, different from Algebric

£ p < 0.05, different from Surrogate
